# Effects of Spray-Drying and Choice of Solid Carriers on Concentrations of Labrasol^®^ and Transcutol^®^ in Solid Self-Microemulsifying Drug Delivery Systems (SMEDDS)

**DOI:** 10.3390/molecules18010545

**Published:** 2013-01-02

**Authors:** Liang Li, Tao Yi, Christopher Wai-Kei Lam

**Affiliations:** State Key Laboratory for Quality Research in Chinese Medicine, Macau Institute for Applied Research in Medicine and Health, Macau University of Science and Technology, Taipa, Macau

**Keywords:** solid SMEDDS, spray-drying, solid carriers, excipients, LC/GC-MS

## Abstract

Solid self-microemulsifying drug delivery systems (SMEDDS) have been used increasingly for improving the bioavailability of hydrophobic drugs. Labrasol^®^ and Transcutol^®^ are used widely as surfactant and solubilizer in the formulation of solid SMEDDS. We investigated the effects of spray-drying and the use of different solid carriers on concentrations of Labrasol^®^ and Transcutol^®^ in solid SMEDDS with scutellarin as the formulated drug. Liquid and gas chromatography tandem mass spectrometry (LC-MS and GC-MS) methods were developed for measuring low concentrations of Labrasol^®^ and Transcutol^®^. In the preparation of solid SMEDDS, lactose, hydroxypropylmethyl cellulose (HPMC) and microcrystalline cellulose (MCC) were used as solid carriers. Judging from the retention ratios of Labrasol^®^ and Transcutol^®^, the droplet size of solid SMEDDS increased after spray-drying of liquid SMEDDS, and concentrations of these excipients decreased after the solidifying procedure. In such reduction, Lactose and HPMC were found to preserve Labrasol^®^ and Transcutol^®^ better than MCC during spray-drying, and the resultant droplet sizes were smaller than that of MCC. Labrasol^®^ and Transcutol^®^ showed good thermal stability at 60 °C degree for 10 days. It can be concluded that spray-drying could increase the droplet size of solid SMEDDS and decreased the concentration of Labrasol^®^ and Transcutol^®^ therein, while water-soluble solid carriers could preserve Labrasol^®^ and Transcutol^®^ better than insoluble carriers in the solid SMEDDS.

## 1. Introduction

Self-microemulsifying drug delivery systems (SMEDDS) have been used increasingly for improving the oral bioavailability of poorly water-soluble drugs [1]. Liquid SMEDDS are a complex system composed, in addition to the drug(s) being formulated, also of excipients including oil, surfactant, and co-surfactant, which are pharmaceutically inactive substances used as carriers or enhancers in the drug system.

Following the addition of solid carriers and a solidifying procedure, solid SMEDDS in stable and convenient dosing forms are prepared [2,3]. Several recent studies have shown that the solidification procedure and choice of different solid carriers could adversely affect the droplet size of the solid SMEDDS and the concentrations of excipients therein [3,4,5,6,7,8,9]. However, there remains a lack of investigations on the effects of different types of solid carriers in solid SMEDDS formulations. It is also important that the solidification procedure should preserve small droplet size for enhancing bioavailability of the formulated drug(s). 

In this study, we have chosen to investigate the effects of spray-drying on the preparation of solid SMEDDS. It is a simple and inexpensive method commonly used for microencapsulation of drug components [2,3,6]. Although spray-drying combines the formulation components and creates a solid drug delivery system, the high drying temperature may affect the stabilities of excipients such as Labrasol^®^ and Transcutol^®^ that are used as solubility enhancers. We assessed the thermal stability of Labrasol^®^ and Transcutol^®^ at 60 °C for 10 days [10]. 

Labrasol^®^ and Transcutol^®^ have been used widely as surfactant and co-surfactant in the preparation of SMEDDS. The former is composed of a mixture of mono-, di- and triglycerides and mono- and di-fatty acid esters of polyethyleneglycol. Its predominant constituents are caprylic acid and capric acid. Labrasol^®^ has been increasingly used because it increases the aqueous solubility of hydrophobic drugs such as piroxicicam, and also improves the bioavailability of hydrophilic drugs such as gentamicin through permeability enhancement [11,12,13,14]. Transcutol^®^ includes 2-(2-ethoxyethoxy)- ethanol. It has been used as an effective solubilizing agent and a permeability enhancer in emulsifying systems [15]. Transcutol^®^ has also been reported to promote the percutaneous penetration of active chemical substances when combined with suitable surfactants such as Labrasol^®^ [16].

The quantitative methods of LC-MS and GC-MS have been applied in the measurement of excipients [17,18], but analysis of these specific excipients added in solid SMEDDS has not been investigated. Considering the low concentrations of Labrasol^®^ and Transcutol^®^ to be measured, it was anticipated that chromatography tandem mass spectrometry should be appropriate. Comparing between gas and liquid chromatography (GC and LC) tandem mass spectrometry (MS), the advantage of GC-MS is that this combined technique has already been applied to fatty acid analysis, whereas for LC-MS, the benefit is that it avoids the derivatization of samples [17,18,19]. We therefore investigated and compared the quantification of Labrasol^®^ and Transcutol^®^ in solid SMEDDS using LC-MS and GC-MS. These methods were also used for studying the thermal stability of Labrasol^®^ and Transcutol^®^ during the spray-drying solidification procedure and storage of solid SMEDDS.

Different solid carriers can affect the release and absorption of drugs in solid SMEDDS, possibly through their influence on droplet size and concentration of excipients independent of drug concentration [2,3,20]. In this study, we assessed the use of three solid carriers in the preparation of solid SMEDDS, including small-molecule lactose (molecular weight = 91 Da) and large-molecule hydroxypropylmethyl cellulose (HPMC, molecular weight > 10 kDa) as water-soluble solid carriers, and microcrystalline cellulose (MCC) as an insoluble carrier. The concentrations of Labrasol^®^ and Transcutol^®^ in solid SMEDDS with the use of these three solid carriers were compared with regard to droplet size and their distribution in the solid SMEDDS. These investigations should be helpful in the characterization and selection of solid carriers.

**Figure 1:**
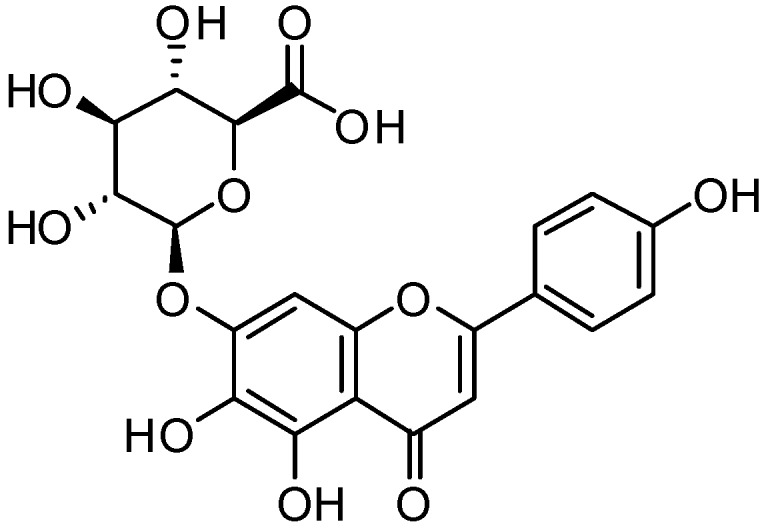
Structure of scutellarin.

For the above studies, scutellarin (Figure 1) extracted from the Chinese herb *Erigerin breviscapus* (Vant.) Hand-Mazz, was chosen as the model drug for our solid SMEDDS [21]. The poor aqueous solubility and low bioavailability of scutellarin have restricted its use in oral preparations, making it an appropriate drug for pharmaceutical enhancement using solid SMEDDS [22]. 

Therefore the objectives of our study were to: (1) prepare scutellarin solid SMEDDS by spray-drying using Labrasol^®^ and Transcutol^®^ and three different solid carriers as excipients, (2) compare changes in droplet size affected by spray-drying and solid carriers; (3) establish LC-MS and GC-MS methods for micro-analysis of Labrasol^®^ and Transcutol^®^ in solid SMEDDS, and (4) investigate the thermal stability and concentration changes of Labrasol^®^ and Transcutol^®^ in solid SMEDDS related to spray-drying and the use of different solid carriers.

## 2. Results and Discussions

### 2.1. Preparation of Liquid and Solid SMEDDS

In the analysis of scutellarin using high performance liquid chromatography (HPLC), the calibration curve was Y = 17.475X − 4.9824 (Y represented peak area of absorbance at 335 nm and X was concentration in μg/mL) with R^2^ = 0.9999. The linearity range was from 0.01–200 μg/mL. The solubility of scutellarin was 0.066 mg/mL in water and 13.13 mg/mL in methanol at room temperature. These confirmed that the drug was poorly soluble in water and well soluble in methanol.

In this study, the formulation of liquid SMEDDS comprised scutellarin, surfactants, co-surfactants and oil. In order to achieve a clear mixture and enhanced solubility of scutellarin in the system, Labrasol^®^, Cremophor^®^ EL and Transcutol^®^ were used as solubilizing and absorption-enhancing excipients in the formulation as a mixed surfactant [23,24,25,26,27,28]. In the preparation of SMEDDS, the solubilities of scutellarin in excipients were first assessed separately. Scutellarin concentrations in Transcutol^®^, Cremophor^®^ EL and Labrasol^®^ were 47.8, 24.0 and 20.9 μg/mL, respectively. Since scutellarin is hydrophobic, its solubility was poor in both Maisine^®^ 35-1 and WL 1349 (at 0.35 and 0.02 μg/mL, respectively). For comparing the effects of different solid carriers in the preparation of solid SMEDDS, we choose lactose as small-molecular and HPMC as higher-molecular water-soluble solid carriers, with MCC as an insoluble solid carrier [6,29]. 

After determining the solubility of scutellarin in specific excipients and its pseudo-ternary phase diagram, the formulation of the liquid SMEDDS was optimized as follows: the oily phase was a combination of Maisine^®^ 35-1 and Labrafac^®^ Lipophile WL 1349 (1:1, w/w), the mixed surfactants were Labrasol^®^ and Cremophor^®^ EL (1:2, w/w), and Transcutol^®^ served as co-surfactant. The mass ratio of mixed surfactants to co-surfactant was 2:1, while that of the oily phase to mixed surfactants was 3:7. The liquid SMEDDS was prepared by dissolving 0.1% (w/w) of scutellarin in the mixture of Transcutol^®^ and Labrasol^®^ in an isothermal water bath at room temperature, then Maisine^®^ 35-1, Labrafac^®^ Lipophile WL 1349 and Cremophor^®^ EL were added. The mixture was mixed by vortexing until a transparent preparation was obtained.

Our optimized conditions for the preparation of solid SMEDDS samples were established as follows: lactose (4 g), HPMC (4 g) and MCC (4 g) were suspended separately in deionized water (200 mL) by magnetic stirring. With constant stirring, the previously prepared liquid SMEDDS (4 g) was added dropwise for 10 min at room temperature into each solid carrier. The prepared suspensions were spray-dried with a B-290 mini spray dryer (Buchi, Flawil, Switzerland). The drying conditions were: aspiration, 90%; inlet temperature, 140 °C; outlet temperature, 66 °C; suspension feeding rate, 5 mL/min. The reconstitution properties, surface characterization, inner physical structure and X-ray powder diffraction of the solid SMEDDS were established as previously described [2,3]. In these analyses, scutellarin in the solid SMEDDS was found to be in amorphous form and not re-crystallized.

### 2.2. Droplet Size of Liquid and Solid SMEDDS

In this study, liquid and solid SMEDDS were characterized in terms of droplet size and polydispersity index (PDI), which are important physical attributes influencing the stability of the emulsion, as well as determining the rate and extent of drug release. Comparing the droplet size and distribution of SMEDDS before and after the spray-drying procedure, it was found that spray-drying increased the droplet size of SMEDDS (Table 1). After the solidification procedure, droplet size of SMEDDS prepared with solid carriers lactose, HPMC or MCC increased 2.0, 5.1 and 4.6 times respectively, resulting in droplet sizes of solid SMEDDS being lactose < HPMC < MCC. Before spray-drying, HPMC liquid-SMEDDS exhibited the smallest droplet size that was half of that with lactose (57.8 *vs.* 121.1 nm). After spray-drying, HPMC solid-SMEDDS droplets seemed to have comparable sizes as those of lactose solid-SMEDDS (294 and 220 nm, respectively). This may suggest that HPMC affected the droplet size more than lactose in the solidifying procedure and the choice of solid carrier is important. Since the droplet size of solid SMEDDS increased the least and was the smallest when lactose was used as the solid carrier, it could be suggested further that during the spray-drying procedure, water-soluble solid carriers with small molecular weight such as lactose may provide better preservation of the droplet size than HPMC as the water-soluble solid carrier with a larger molecular weight, and MCC which is an insoluble solid carrier. 

**Table 1:** Droplet sizes and PDI of liquid and solid SMEDDS using lactose, HPMC and MCC as solid carriers. Results are expressed as mean ± SD of vlaues from triplicate experiments.

	Samples	Droplet Size(nm)	PDI
1	Lactose L-SMEDDS	112.1 ± 7.6	0.276 ± 0.031
2	Lactose S-SMEDDS	220.0 ± 10.7	0.494 ± 0.036
3	HPMC L-SMEDDS	57.8 ± 4.9	0.398 ± 0.045
4	HPMC S-SMEDDS	294.1 ± 9.7	0.592 ± 0.068
5	MCC L-SMEDDS	93.6 ± 4.5	0.418 ± 0.044
6	MCC S-SMEDDS	429.0 ± 15.6	0.681 ± 0.079

### 2.3. Development and Validation of GC-MS Method

**Figure 2:**
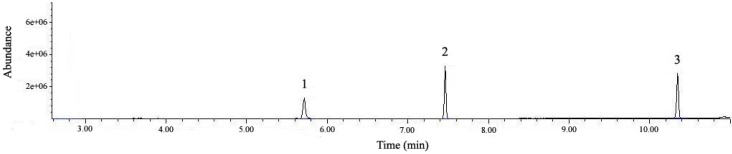
Total ion chromatogram (TIC) of a standard mixture of Labrasol^®^ and Transcutol^®^ by GC-MS. Peak 1: Transcutol^®^, Peak 2: caprylic acid in Labrasol^®^, and Peak 3: capric acid in Labrasol^®^.

For the quantification of Labrasol^®^ and Transcutol^®^ using GC-MS, a standard 30-meter fused silica column was used. The conditions of injection volume, split ratio and temperature program were optimized to achieve good peak separations. Previous analysis of fatty acids by gas chromatography comprised basically the derivatization of the relatively high polarity acids into non-polar compounds, among which the fatty acid methyl esters (FAMEs) were universally used [30]. The acid conditions of Labrasol^®^ or Transcutol^®^ in anhydrous methanol were optimized to pH = 3.4 for 30 min. The derivatization products of Labrasol^®^ were detected in GC-MS as methyl caproate (*m/z* 186.3) at 10.31 min and methyl caprylate (*m/z* 158.2) at 7.43 min, while 2-(2-ethoxyethoxy)-ethanol (*m/z* 134.2) in Transcutol^®^ was detected in its prime structure at 5.60 min (Figure 2). The retention times and fragment ions of Labrasol^®^ and Transcutol^®^ are shown in Table 2. 

Both Labrasol^®^ and Transcutol^®^ were quantified using a seven-point calibration from a set of standard solutions. Based on the relative standard deviation (RSD), the limit of detection (LOD) was set at signal-to-noise ratio of 3:1, and for the limit of quantification (LOQ), the ratio of 10:1. We found that the LOD was 2.5, 3.2 and 0.80 ng/mL for methyl caproate, methyl caprylate and 2-(2-ethoxyethoxy)-ethanol, respectively, and the corresponding LOQ were 4.3, 4.4 and 0.99 ng/mL. The standard curves and R^2^ values are shown in Table 3. Each sample was tested for at least three times and RSD of every data acquired was less than 5%. The RSD of retention times of methyl caproate, methyl caprylate and 2-(2-ethoxyethoxy)-ethanol were 0.035, 0.041 and 0.017%, respectively. The corresponding precision values were 2.4, 4.0 and 3.8%, and bias values were ±3.2, 2.5 and 2.6%.

**Table 2:** Analytical parameters for the identification of Labrasol^®^ and Transcutol^®^ by GC-MS with EI ion source, and LC-MS with ESI ion source. *t_R_* denotes retention time, *M_r_* denotes relative molecular mass.

Sample	*t_R_*(min)	*M_r_*(*m/z*)	Precursor and product ion of EI in GC-MS, ESI negative in Labrasol^®^ and positive in Transcutol^®^ in LC-MS	Compound
Labrasol^®^ by GC-MS	10.31	186.3	186[M]^+^, 155[M–O–CH_3_]^+^, 143[M–C_3_H_7_]^+^, 87[M–C_7_H_15_]^+^, 74[M–C_8_H_16_]^+^	Methyl caproate
Labrasol^®^ by GC-MS	7.43	158.2	158[M]^+^,127[M–O–CH_3_]^+^,115[M–C_3_H_7_]^+^, 87[M–C_5_H_11_]^+^,74[M–C_6_H_12_]^+^	Methyl caprylate
Transcutol^®^ by GC-MS	5.60	134.2	134 [M]^+^,133[M−H]^+^, 117[M−OH]^+^, 104[M–O–CH_2_]^+^,72{[M−H]–O–C_2_H_4_–OH}^+^	2-(2-Ethoxyethoxy)-ethanol
Labrasol^®^ by LC-MS	21.16	172.3	171.1[M−H]^−^, 130.1[M–C_2_H_6_]^−^, 127.1[M–COOH]^−^, 87.1[M–C_6_H_13_]^−^	Capric acid
Labrasol^®^ by LC-MS	17.13	144.2	143.1[M−H]^−^,130.2[M–CH_2_]^−^, 98.2[M–COOH]^−^,56.3[M–C_3_H_6_–COOH]^−^	Caprylic acid
Transcutol^®^ by LC-MS	7.11	134.2	135.2[M+H]^+^,117.3[M–OH]^+^, 103.2[M–CH_2_–OH]^+^, 73.1[M–O–C_2_H_4_–OH]^+^	2-(2-Ethoxyethoxy)-ethanol

**Table 3:** Standard curves and linearity ranges of Labrasol^®^ and Transcutol^®^ analyses using GC-MS and LC-MS.

Sample	Standard Curve *	*R^2^*	Linearity Range (ng/mL)
Methyl caproate by GC-MS	Y = 3 × 10^7^X + 249096	0.9997	871–4.33
Methyl caprylate by GC-MS	Y = 3 × 10^7^X + 535589	0.9993	878–4.45
2-(2-Ethoxyethoxy)-ethanol by GC-MS	Y = 247655X − 450994	0.9990	197–0.99
Capric acid by LC-MS	Y = 9 × 10^7^X + 5 × 10^6^	0.9991	886–8.90
Caprylic acid by LC-MS	Y = 10^6^X + 278620	0.9990	911–4.61
2-(2-Ethoxyethoxy)-ethanol by LC-MS	Y = 170824X + 852542	0.9991	197–4.92

* Y represented peak area of absorbance at 335 nm and X was concentration in μg/mL.

### 2.4. Development and Validation of LC-MS Method

**Figure 3:**
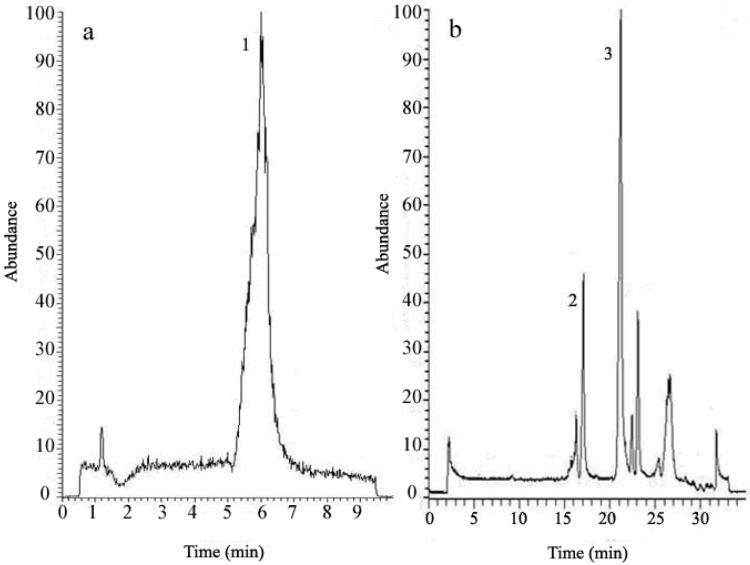
Total ion chromatogram (TIC) of the standard solutions of Transcutol^®^ and Labrasol^®^ detected by LC-MS. Peak 1 in chromatogram (**a**): Transcutol^®^, Peaks 2 and 3 in chromatogram (**b**): caprylic acid and capric acid in Labrasol^®^, respectively.

In this analysis, a standard C_18_ reverse phase column with particle size of 5 μm was used, but the peak resolution was poor. Therefore particle size of 3.5 μm was employed to acquire a satisfactory separation. In the analysis of both Labrasol^®^ and Transcutol^®^, the column temperature, gradient elution procedure and injection volume were optimized. Since Labrasol^®^ and Transcutol^®^ did not show substantial absorbance in both the visible and ultraviolet spectra, LC-MS was employed for detecting target peaks [31]. In the MS detection, the negative mode was used for analysis of fatty acids in Labrasol, while the positive mode was employed in the Transcutol^®^ detection [32]. As shown in Figure 3, capric acid (*m/z* 172.3) was eluted at 21.16 min and caprylic acid (*m/z* 144.2) at 17.13 min in the standard solution of Labrasol^®^, while 2-(2-ethoxyethoxy)-ethanol (*m/z* 134.2) in Transcutol^®^ was eluted at 7.11 min under another gradient separation program. The identification of the precursor and product ions in LC-MS with ESI ion source is shown in Table 2 above. For each compound, a standard curve was generated integrating peak area of seven stepwise diluted injections, each from triplicate analyses. The LOD was determined to be 4.1, 17.2 and 2.8 ng/mL for capric acid, caprylic acid and 2-(2-ethoxyethoxy)-ethanol, while corresponding LOQ were 8.9, 4.6 and 4.9 ng/mL. Precision values were 2.2, 3.5 and 4.9%, and bias values were ±1.7, 2.4 and 2.6%, and RSD of each average peak area and retention time was less than 5%. Table 3 above shows the R^2^ values and standard curves of Labrasol^®^ and Transcutol^®^ using LC-MS. 

### 2.5. Thermal Stability of Labrasol^®^ and Transcutol^®^

**Figure 4:**
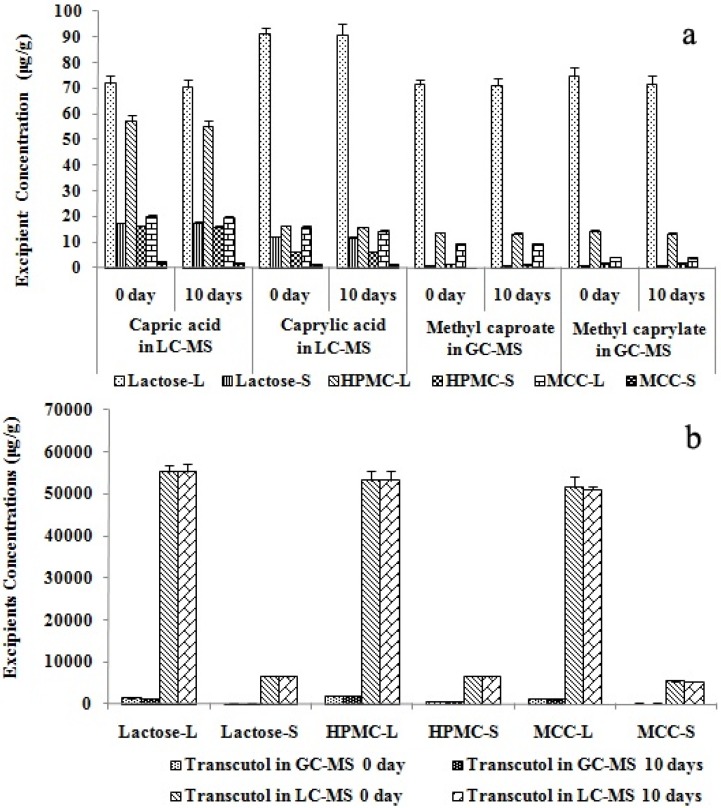
Thermal stability of Labrasol^®^ and Transcutol^®^ assessed by LC-MS and GC-MS. Labrasol^®^ (**a**) and Transcutol^®^ (**b**) concentrations measured by LC-MS and GC-MS are shown (n = 4, *p* > 0.05). The 0-day concentrations were measured before excipients underwent the thermal stability test, while 10-day concentrations were for concentrations of excipients at 10 days after commencement of the tests.

To further assess the thermal stability of Labrasol^®^ and Transcutol^®^ during the storage of solid SMEDDS, concentration changes in these excipients in liquid and solid SMEDDS were measured before and after storage at 60 °C for 10 days. Results showed that Labrasol^®^ and Transcutol^®^ concentrations did not differ (Figure 4, all *p* > 0.05), and the drug did not recrystallize.

It can be concluded that Labrasol^®^ and Transcutol^®^ are thermally stable at 60 °C for 10 days in liquid and solid SMEDDS. Therefore room temperature storage of these formulations over a short period of time should not affect the stability of the two excipients therein. These findings should be adequate for the general usage of liquid and solid SMEDDS under routine therapeutic circumstances, although long-term thermal stability at more unfavorable temperatures and periods might need further investigation.

### 2.6. Effect of Spray-Drying on Labrasol^®^ and Transcutol^®^ in Solid SMEDDS

**Figure 5:**
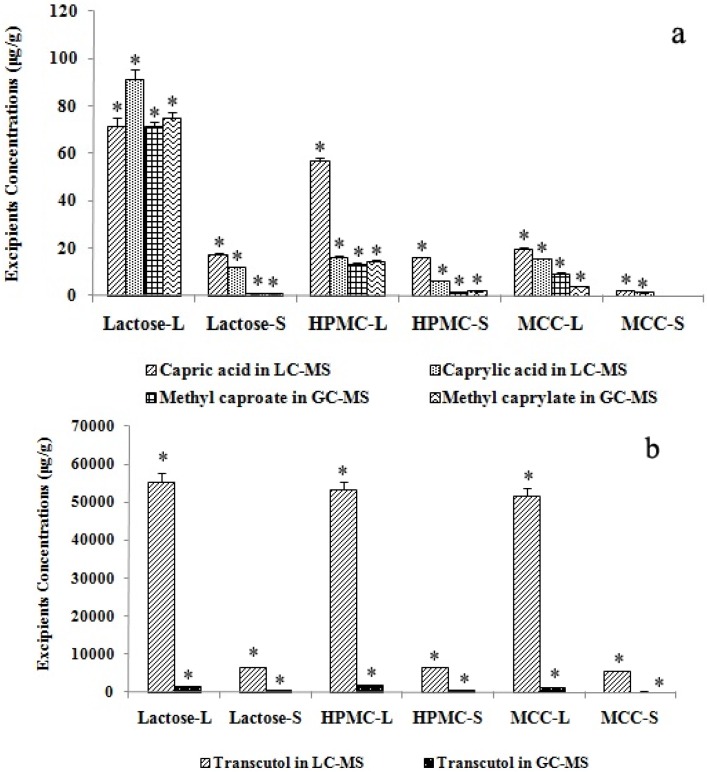
Concentrations of Labrasol and Transcutol in SMEDDS of scutellarin. Labrasol (**a**) and Transcutol (**b**) concentrations were analyzed by LC- and GC-MS (n = 4, * *p* < 0.05). Lactose-L, HPMC-L and MCC-L represent liquid SMEDDS containing lactose, HPMC and MCC; while lactose-S, HPMC-S and MCC-S are for solid SMEDDS with lactose, HPMC and MCC as solid carriers. * denotes *p* < 0.05.

Figure 5 shows the final calculated concentrations of Labrasol^®^ and Transcutol^®^ measured by GC-MS and LC-MS. The concentrations of methyl caproate and methyl caprylate in the GC-MS analysis corresponded to those of capric acid and caprylic acid in the LC-MS determination. 

It can be clearly seen that spray-drying caused reduction of excipient concentrations in the solid SMEDDS. Such reduction in the concentration of solubilizer and co-surfactant (Labrasol^®^ and Transcutol^®^) may have a lowering effect on the saturation solubility of the formulated drug (scutellarin). The two analytical methods were both reliable, but since GC-MS involved a derivatization process and the ionization mode of ESI in LC-MS was softer than EI in GC-MS analysis, the peak areas in the TIC of LC-MS were larger than those detected by GC-MS. This suggests that the LC-MS method might be more accurate in the analysis of the two excipients. 

### 2.7. Effect of Different Solid Carriers on Labrasol^®^ and Transcutol^®^ in Solid SMEDDS

To compare the effect of different solid carriers on Labrasol^®^ and Transcutol^®^ concentration by LC-MS and GC-MS, retention ratio (w/w) was calculated by dividing the concentration of solid SMEDDS by the liquid SMEDDS concentration. Figure 5a shows that in the LC-MS analysis of Labrasol^®^, the concentration sequence of capric acid and caprylic acid in the three liquid and solid SMEDDS samples with different solid carriers were lactose > HPMC > MCC (*p* < 0.05). However, in the GC-MS determination, the concentrations of methyl caproate and methyl caprylate were also lactose > HPMC > MCC (*p* < 0.05), but the difference between lactose and HPMC was smaller than that in LC-MS, and lactose concentration was higher than that in HPMC. These findings could be due to the thickening property of HPMC: it might combine with Labrasol^®^ to adhere to part of the LC column, leading to the lower concentrations in HPMC than in lactose. Table 4 shows that the retention ratios of Labrasol^®^ in LC-MS analysis were HPMC > lactose > MCC (*p* < 0.05). However, the sequence was HPMC > MCC > lactose (*p* < 0.05) in GC-MS. This variation between LC-MS and GC-MS analyses was probably due to the derivatization procedure when Labrasol^®^ was not totally evaporated in GC analysis compared to the LC procedure.

Therefore, it could be implicated that both the capric acid and caprylic acid were better dissolved in lactose and HPMC than in MCC, this should be due to the better water-solubility of lactose and HPMC than the insoluble solid carrier MCC. On the other hand, the retention ratio of Labrasol^®^ was the highest when HPMC was the solid carrier during the spray-drying procedure, this can be explained by the better thickening property of HPMC. 

The results of analysis of Transcutol^®^ in solid SMEDDS with different solid carriers were similar using LC-MS and GC-MS methods (Figure 5b). The concentrations were HPMC > lactose > MCC (*p* < 0.05) for both LC-MS and GC-MS. Also, the sequences of retention ratios (w/w) were the same for LC and GC-MS. These results are related to the properties of the three solid carriers. Different from MCC, lactose and HPMC are water-soluble solid carriers. In addition, HPMC is also better dispersible in water, therefore HPMC is more suitable for preparing solid SMEDDS considering the spraying drying retention ratio of Transcutol^®^ and Labrasol^®^ [32,33].

In our pilot study, there was a correlation between Labrasol^®^ and Transcutol^®^ concentrations and the recovery of scutellarin after spray-drying. Different solid carriers also exerted significant effects on drug encapsulation after spray-drying. For example, scutellarin contents of 56.5, 10.3, 15.7 μg/g in liquid SMEDDS, solid SMEDDS with HPMC, and solid SMEDDS with lactose as solid carriers showed that there was a difference between the use of HPMC and lactose (both water soluble carriers) on the recovery of scutellarin after spray-drying, suggesting that solid carriers influenced drug encapsulation.

Another point of discussion is that spray-drying should be sensitive to viscosity of the spray solution especially when high molecular weight HPMC is used. We have previously prepared solid SMEDDS using HPMC as the solid carrier [2]. Since the liquid-SMEDDS for spray-drying contained a lot of water, the addition of high molecular weight HPMC increased the viscosity of the system but did not affect the successful preparation of the solid SMEDDS. Our reconstitution study also showed that the solid SMEDDS could form micro-emulsions, thereby changing the drug releasing rate from immediate release to slow release.

**Table 4:** Retention ratios of Labrasol^®^ and Transcutol^®^ in solid SMEDDS after spray- drying determined by LC- and GC-MS. Ratios are expressed in mean ± SD from quadruplicate analyses.

Solid carriers	Labrasol^® ^by LC-MS		Labrasol^®^ by GC-MS		Transcutol^®^ by LC-MS	Transcutol^®^ by GC-MS
	Capric acid (%)	Caprylic acid (%)	Methyl caproate(%)	Methyl Caprylate(%)	2-(2-Ethoxy-ethoxy)-ethanol(%)	2-(2-Ethoxy-ethoxy)-ethanol(%)
**Lactose**	24.25 ± 2.31	12.91 ± 1.11	0.95 ± 0.18	0.79 ± 0.19	11.77 ± 1.02	24.51 ± 1.70
**HPMC**	28.16 ± 1.70 ^a^	38.19 ± 2.94 ^a^	9.86 ± 1.53 ^a^	12.45 ± 1.13 ^a^	12.36 ± 1.41 ^a^	33.16 ± 2.63 ^a^
**MCC**	11.16 ± 1.03 ^b^	8.15 ± 1.32 ^b^	1.07 ± 0.27 ^b^	6.06 ± 0.42 ^b^	10.45 ± 0.33 ^b^	18.53 ± 1.33 ^b^

^a^* p* < 0.05, when compared with the parameters of lactose and MCC by the ANOVA test. ^b^* p* < 0.05, when compared with the parameters of lactose and HPMC by the ANOVA test.

## 3. Experimental

### 3.1. Materials and Samples

Maisine^®^ 35-1 (glyceryl monolinoleate), Labrafac^®^ Lipophile WL 1349 (medium chain triglycerides), Labrasol^®^ (caprylocaproyl macrogolglycerides) and Transcutol^®^ HP (diethylene glycol monoethyl ether) were purchased from Gattefossé Co. Ltd. (Cedex, France). Cremophor^®^ EL (polyoxy l35 castor oil) was supplied by BASF China Co. Ltd. (Shanghai, China). Lactose (Flowlac^®^ 100) was obtained from Meggle Co. Ltd. (Wasserburg, Germany). HPMC was purchased from Colorcon Coating Technology Ltd. (Shanghai, China), and MCC (microcrystalline cellulose) from Asahi Kasei Microsystems Co. Ltd. (Tokyo, Japan). A crude sample of scutellarin with a purity of 86% was obtained from Wangfang Co. Ltd. (Yunnan, China), and the standard sample of scutellarin was purchased from the State Food and Drug Administration of China (SFDA, Shanghai, China). HPLC grade methanol, acetonitrile and acetic acid were obtained from Merck Co. Ltd. (Darmstadt, Germany). Deionized water was purified using an Ultra-purification Water System (Millipore Co. Ltd., Bedford, MA, USA). 

### 3.2. Preparation of Liquid and Solid SMEDDS

Solubility of scutellarin in the following vehicles, including oils (Maisine^®^ 35-1 and WL 1349), surfactants (Labrasol^®^ and Cremophor^®^ EL), auxiliary solvent (Transcutol^®^), distilled water, and methanol at room temperature were determined by the shake flask method. In this method, an excess amount of scutellarin (approximately 1 g) was added to 1 mL of the different vesicles. After sealing the vials of mixtures, Barnstead Speed Control 3760 mixer (Thermo-Fisher Scientific Inc., San Jose, CA, USA) was used to vortex the mixtures at maximum speed for 10 min, and kept for 48 h at 25 °C in a shaking water bath to facilitate solubilization. The mixtures were centrifuged at 3,000 *g* for 15 min using the Beckman Allegra X-15R centrifuge (Beckman Coulter Inc. Fullerton, CA, USA) to remove un-dissolved scutellarin. The supernatants were collected and diluted with methanol for quantification of scutellarin by HPLC. 

Scutellarin was analyzed using the Agilent 1200 HPLC (Agilent Technologies, Santa Clara, CA, USA) consisting of a quaternary pump, on-line degasser, well-plate autosampler, thermostatic column compartment and diode-array detector, using a 250 mm × 4.6 mm I.D. particle size 5 μm C18 column (Waters Technologies Ltd., Wexford, Ireland) with a pump flow of 1 mL/min^−1^, column temperature at 40 °C and injection volume of 5 μL. The mobile phase was a gradient of solvent A (0.1% phosphoric acid) and solvent B (acetonitrile), applied as follows: 0–10 min, 20% B; 10–20 min, 20%–100% B. Scutellarin was eluded at 7.5 min as monitored via absorbance at 335 nm using a diode-array detector. Scutellarin in SMEDDS samples was also analyzed by this method. 

### 3.3. Droplet size and Distribution of Liquid and Solid SMEDDS

Liquid SMEDDS (100 μL) or solid SMEDDS (100 mg) was added to 10 mL deionized water in a volumetric flask and the mixture was agitated at 400 rpm for 30 min. Droplet size was determined using a Malvern Nano ZS 90 laser droplet size analyzer (Malvern Instruments Ltd., Worcestershire, UK). The detection range was from 2 to 1,000 nm and PDI was used for measuring the width of distribution. Each sample was analyzed in triplicate.

### 3.4. Thermal Stability Test

The thermal stability test [34] was employed for investigating the stability of Labrasol^®^ and Transcutol^®^ during the storage of SMEDDS. All liquid and solid SMEDDS samples were placed in a Shutzart thermostatic incubator (Membert GmbH & Co. KG, Schwabach, Germany) set at 60 ± 1 °C in the dark for 10 days. Labrasol^®^ and Transcutol^®^ were measured before and after the incubation. Triplicate experiments were performed on each sample.

### 3.5. Standard Solutions of Labrasol^®^ and Transcutol^®^

Labrasol^®^ standards were prepared from a stock solution containing Labrasol^®^ (10 μg) in methanol (10 mL). The calibration standards were serially diluted from the stock solution with methanol to 4 ng/mL. Transcutol^®^ calibration standards were prepared from diluting a stock solution (10 μg/mL) with methanol to 0.8 ng/mL. All calibration standards were freshly prepared before the experiments.

### 3.6. Sample Preparation for Analysis of Labrasol^®^ and Transcutol^®^

Separately, solid SMEDDS (10.0 mg) prepared using the solid carrier of lactose, HPMC or MCC were added to methanol (1.0 mL). For liquid SMEDDS, samples (100 μL) were diluted with methanol (1 mL). The solutions or mixtures were then vortexed with a mixer for 5 min and centrifuged at 4,500 *g* for 10 min. The supernatants were filtered through a 0.45 μm membrane filter before injection into the LC-MS and GC-MS for analysis of Labrasol^®^ and Transcutol^®^. Samples from the thermal stability test were treated with the same procedure. 

### 3.7. GC-MS Analysis of Labrasol^®^ and Transcutol^®^

The analysis was performed on an Agilent 6890N series gas chromatography system using model 5973 mass selective detector with electron impact (EI) ion source, coupled with 7683B series injector using a J&W DB-5MS 30 m × 0.25 mm I.D., 0.25 μm film thickness fused silica column. A constant column flow of 1 mL/min helium was applied. The capillary split/splitless inlet was maintained at 200 °C with a split ratio of 1:9. After injecting the samples (10 μL) into to the system, the initial oven temperature was set at 50 °C, followed by a programmed temperature ramp of 10 °C/min to 150 °C which was held for 1 min. The MS signals were scanned from *m/z* 40–200 and in the selected ion monitoring (SIM) mode applying the mass ions of *m/z* 134, 158 and 186 for detection. All GC-MS quantification was based on a peak area ratio of the SIM signal of the analyte, and the scan signal was used to verify the identities of chromatographic peaks. The source temperature was kept at 240 °C with the transfer line of 280 °C and quadruple at 150 °C. A representative chromatogram of the standard mixture is shown in Figure 2. 

### 3.8. LC-MS Analysis of Labrasol^®^ and Transcutol^®^

A HPLC system (Thermo Fisher Scientific Inc.) consisting of an Accela auto-sampler and Accela pump interfaced with a TSQ Quantum triple-quadrupole mass spectrometer was used. The chromatography column was a Waters Sunfire C-18 HPLC column with particle size 3.5 μm, 150 mm × 2.1 mm I.D. The mobile phase comprised a gradient of solvent A (0.2% acetic acid) and solvent B (acetonitrile) at a flow rate of 200 μL/min, with the sample injection volume of 10 μL and column temperature of 20 °C. The elution conditions for Transcutol^®^ were 0–5 min, 5%–10% B; 5–10 min, 10%–20% B, and for Labrasol^®^ 0–5 min, 20% B; 5–25 min, 20%–60% B; 25–30 min, 60%–100% B. 

For MS analysis, the electro-spray ionization (ESI) interface was tested at 4,000 V by scanning between *m/z* 30–500 per second. Nitrogen was used as spray gas, curtain gas and heater gas, and set respectively at the flow rates of 0.5, 1.5 and 6.0 mL/min, while the mass spectrometer was set automatically during the MS tune procedure. The MS signal of Labrasol^®^ was collected in SIM mode with the quasi-molecular ions of *m/z* 143 (caprylic acid) and 171 (capric acid) in negative mode. For Transcutol^®^, the MS signal was analyzed at *m/z* 135 (Transcutol^®^) in positive mode. The scan signal was used to verify the identities of the chromatographic peaks, while all LC-MS quantification was based on a peak area ratio of the SIM signal of the analyte. Figure 5 shows the chromatograms obtained from liquid SMEDDS sample mixtures analyzed by LC-MS. 

### 3.9. Statistical Analysis

All results are expressed as mean ± SD. Data from different solid carriers were compared for statistical significance by one way analysis of variance (ANOVA). Statistical analyses were performed using SPSS software version 4.0 (SPSS, Chicago, IL, USA).

## 4. Conclusions

Self-microemulsifying drug delivery systems have been increasingly used for improving the oral bioavailability of poorly water-soluble drugs, but only a limited amount of study has been conducted on the effects of spray-drying and choice of solid carriers on concentrations and stability of surfactant and co-surfactant (e.g., Labrasol^®^ and Transcutol^®^) in solid SMEDDS. Using LC-MS and GC-MS analyses which can reliably measure Labrasol^®^ and Transcutol^®^ at low concentrations, we found that the spray-drying procedure in the preparation of solid SMEDDS could decrease the concentrations of Labrasol^®^ and Transcutol^®^ therein. Our observed differences in the effects of lactose, HPMC and MCC on Labrasol^®^ and Transcutol^®^ concentrations and the droplet size of solid SMEDDS suggested that different solid carriers could affect the excipients in the solidifying procedure causing increase in droplet size. An appropriate choice of solid carrier based on its water solubility and molecular weight could optimize droplet size, influence the concentrations of excipients, and enhance the bioavailability of drugs in solid SMEDDS. 
